# Human tissue lead (Pb) levels and amyotrophic lateral sclerosis: a systematic review and meta-analysis of case–control studies

**DOI:** 10.1007/s10072-022-06237-y

**Published:** 2022-07-09

**Authors:** Cristiano Farace, Giovanni Fiorito, Andrea Pisano, Federica Etzi, Angela Sabalic, Grazia Fenu, Yolande Asara, Giuliana Solinas, Roberto Madeddu

**Affiliations:** 1 Department of Biomedical Sciences-Histology, University of Sassari, Viale S. Pietro 43b, 07100 Sassari, Italy; 2 National Institute of Biostructure and Biosystem, Rome, Italy; 3 Department of Biomedical Science-Hygiene, University of Sassari, Sassari, Italy; 4 School of Public Health, Imperial College, London, UK

**Keywords:** Amyotrophic lateral sclerosis (ALS), Lead (Pb), Blood, Plasma, Cerebrospinal fluid (CSF), Meta-analysis

## Abstract

**Aim:**

To combine the current scientific literature evidence and elucidate the differences of lead (Pb) bioaccumulation in human tissues by comparing amyotrophic lateral sclerosis (ALS) patients and healthy controls.

**Methods:**

We systematically searched for case–control studies on the association of Pb levels with ALS, in human cells, tissues, and body fluids (nervous tissue, muscle, blood, cerebrospinal fluid, urine, skin appendages). Then, we performed a meta-analysis for all the tissues in which at least five case–control studies were available: whole blood (9 studies), serum/plasma (5 studies), and cerebrospinal fluid (CSF) (6 studies). Differences between cases and controls were evaluated using standardized mean difference, and combined estimates were derived using random effect maximum likelihood (REML) meta-analyses.

**Results:**

Among 1734 records, we identified 46 full-text studies, of which 14 case–control studies met the meta-analysis inclusion criteria. We found higher Pb levels in ALS cases than controls in blood (standardized mean difference (SMD) = 0.61; 95% confidence interval (CI) 0.20, 1.01; *p* = 0.003), plasma/serum (SMD = 0.27; 95% CI − 0.16, 0.70; *p* = 0.26), and CSF (SMD = 0.53; 95% CI − 0.09, 1.15; *p* = 0.09).

**Conclusions:**

This work provides further evidence of the association between Pb bioaccumulation and ALS in body fluids. The lack of association studies in solid tissues did not allow a robust meta-analysis. Future prospective studies are needed to clarify the causality in the association of Pb bioaccumulation with ALS.

## Introduction

Amyotrophic lateral sclerosis (ALS) is an incidence-increasing neurodegenerative disease in adulthood with no cure, which affects the whole compartment of cortical, bulbar, and spinal motor neurons, causing the irreversible collapse of the nervous system control of the voluntary muscle activity, muscular atrophy, and death [1, 2]. The onset and progression of ALS rely on a multifactorial etiology which includes multiple genetic and environmental factors and their mutual interactions, and both act either in familiar (fALS) or sporadic (sALS) forms of the pathology, albeit with different molecular mechanisms and patterns [3, 4].

Clinically, fALS and sALS patients undergo similar diagnostic procedures (i.e., El Escorial criteria) and pharmacological treatments. However, treatments cannot stop the disease progression, but they can only delay the worsening of the disease. On the other hand, the patients’ age of onset and diagnosis seems to be globally decreasing, with a difference of 5–10-year anticipation in fALS cases in relation to sALS patients, probably attributable to the effect of Mendelian ALS-related gene variants more frequent in fALS rather than in sALS, and not merely to ascertainment bias such the increasingly early diagnosis in healthy individuals with relatives affected by ALS [5]. Hence, the genetic burden plays a key role in fALS patients, accounting for 5–10% of the ALS population. Conversely, most ALS patients (90–95%) suffer from the sporadic form of disease dependent on unknown genetic variants and environmental factors, whose exact etiology and underlying pathogenic cellular and molecular mechanism are still to solve [4].

Along with the environmental factors responsible for sALS, chemicals, toxins (i.e., β-methylamino-L-alanine), viruses, and others have been counted [6], diminishing the environmental risk may lead to a decrease in ALS incidence and progression. These and other factors may influence ALS physiopathology, including sex, age, spinal vs bulbar onset, and tissue concentrations of persistent pollutants. Therefore, they are interpretable as risk factors and survival predictors [3]. Metals, particularly heavy metals, are persistent environmental factors supposed to be primarily involved in the etiology and pathogenic mechanism of sALS, but they also seem to play a role in fALS disease. Notably, heavy metal biomagnification in exposed living species and their food chains leads to high bioaccumulation in organisms, including humans, with health concerns for the most susceptible individuals. Most ALS associations have been found for lead (Pb), a lipophilic metal with no function in the human body which accumulates in tissues and organs following chronic and/or acute exposure, including the nervous system, due to its recognized high ability to cross the blood–brain barrier [7–9].

Recently, we critically reviewed the ALS–Pb relationship through a systematic review of the scientific literature of the last decade (2011–2020), giving a complete picture of the most recent epidemiological, clinical, and experimental evidence of the pathogenic role of Pb in ALS, reporting also a potential ALS pathogenic mechanism of Pb involving TAR DNA-binding protein misfolding and accumulation [10]. In that work, we retrieved and included only research articles in the manuscript, without referencing reviews, meta-analyses, and other types of manuscripts, categorized separately. From the last task, we noticed a lack of specific meta-analyses considering the Pb bioaccumulation and quantification in human matrices of ALS patients and healthy controls. Most meta-analyses combined Pb levels in human samples, mainly blood, with other indirect Pb–exposure parameters, such as personal questionnaires, environmental and occupational Pb levels, and others. However, the human metal biomonitoring through direct measurement of the effective Pb concentration in human biological tissues still represents the most powerful procedure to evaluate the current Pb exposure and body absorption. This is particularly relevant when there are discrepancies in Pb bioaccumulation, attributable to a different Pb genetic susceptibility among individuals living in a similar environment. Moreover, our recent systematic update (2011–2020) lacks meta-analyses since it did not include the scientific literature before 2011. For all these reasons, a critical revision of the clinical information on ALS derived from gold standard quantitative analysis of Pb bioaccumulation in human tissues is needed. Here, we critically performed a systematic review of case–control studies published at any time evaluating the Pb levels in any human matrix, such as isolated cells, solid tissues, and body fluids derived from ALS patients and healthy donors. We performed three different meta-analyses of Pb levels in ALS case–control studies considering eligible studies: on whole blood, serum/plasma, and cerebrospinal fluid (CSF), using a restricted maximum likelihood estimation of the random effects (REML) model in a frequentist framework.

## Methods

### Systematic review method

The research was performed following the Preferred Reporting Items for Systematic Reviews and Meta-Analyses (PRISMA) statement [11]. Preliminarily, four authors independently collected published studies on human ALS–Pb relationship, up to February 2022, in PubMed and Scopus databases. The search profile consisted of “lead AND amyotrophic AND lateral AND sclerosis” with 1405 preliminary results on PubMed and 3144 preliminary results on Scopus; and “metals AND amyotrophic AND lateral AND sclerosis” with 1329 preliminary results on PubMed and 764 preliminary results on Scopus.

The reference list of the main reviews on heavy metals and trace elements in ALS patients was also examined to identify additional papers that had been missed. This last task confirmed the lack of specific meta-analysis considering the presence of Pb in human tissues, which is the main aim of this work. Duplicate studies among databases were removed to obtain the first set of papers that focused on Pb and ALS. For search profiles, all titles of the first set of papers were examined and abstracts read of those may be being relevant.

**Fig. 1:**
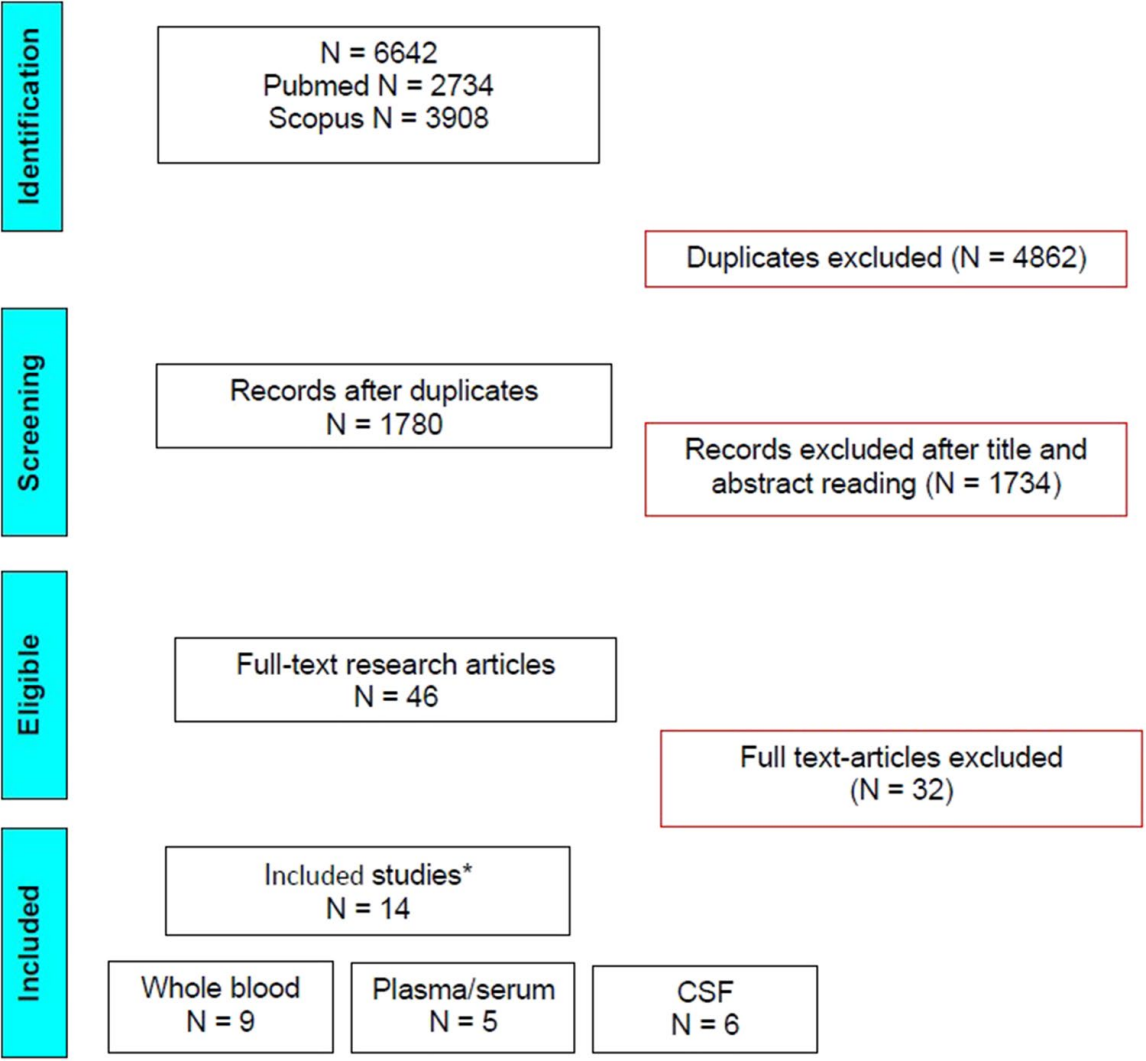
PRISMA flow chart. The flow chart illustrates the number of articles included and excluded at various steps. *The sum of the studies for blood, plasma/serum, and CSF (cerebrospinal fluid) is not equal to the final number of the included studies since four studies investigated two or more matrices

Eligible criteria were case–control studies with results on Pb levels, given as mean ± SD or median and interquartile range, measured in any type of tissue of patients with ALS and healthy donors (controls). The eligibility and final inclusion of each manuscript were assessed independently by two groups of authors, and disagreements were deeply discussed between groups to reach a common conclusion. Meta-analysis of selected tissues was performed as described in following paragraph (“Meta-analysis statistical method” section) if the number of studies for each tissue was ≥ 5. An overview of the selection process is depicted by the PRISMA flow diagram in Fig. 1.

### Meta-analysis statistical method

Combined standardized mean differences between ALS cases and healthy controls and 95% CIs were calculated through a REML meta-analysis to avoid possible heterogeneity between studies [12]. Since, in some studies, the authors summarized the data by reporting the sample medians and one or both of (i) the minimum and maximum values and (ii) the first and third quartiles, without reporting the means or standard deviations, we have estimated means and standard deviations for ALS cases and healthy controls from medians and quartile values according to Weir and colleagues [13]. All the analyses were performed using the R software for statistical analyses, and the meta-analyses were performed using the R package *metafor* [14].

## Results

After an in-depth systematic review of the literature, a total of nine studies with Pb measured in whole blood [15–23], five studies with Pb measured in plasma [18, 24, 25] or serum [15, 26], and six studies with Pb measured in CSF [18, 24, 26–29] met the inclusion criteria and were considered eligible for meta-analysis. Hence, we were able to perform a robust meta-analysis of Pb levels on a total of 409 ALS cases and 575 controls for whole blood, 64 ALS cases and 67 controls for plasma/serum, and 114 ALS cases and 130 controls for CSF. Other studies met the inclusion criteria for other tissues: bone (tibia and patella), urine, skeletal muscle, and nails. However, due to the low number of studies representative of these specific tissues, they were considered eligible for the systematic review but not included in the meta-analysis.

### Blood Pb in ALS patients and healthy donors

#### Whole blood

**Fig. 2:**
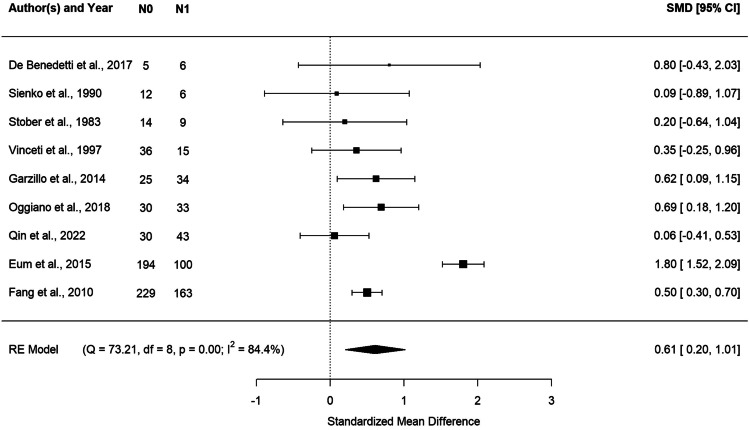
Forest plot: meta-analysis of nine studies on blood samples. Each study is represented with a square centered at the point estimate of the standardized mean difference, with a horizontal line (whiskers) extending on either side of the square, representing the 95% confidence interval of the point estimate. The area of the square is proportional to the corresponding study weight. Combined estimate and 95% confidence interval is represented with a black diamond

The results of the meta-analysis on whole blood Pb are described in Fig. 2 via forest plot. We found significantly higher levels of Pb in the blood of ALS cases compared to controls. The average standardized difference was 0.61, with a 95% CI of [0.20, 1.01] and *p* value = 0.003.

We did not find evidence of heterogeneity in the effect among studies considering the Cochran’s *Q* (*Q* = 73.21, df = 8, *p* < 0.0001) and the *I*^2^ statistics (*I*^2^ = 84.4%) (Fig. 2).

#### Plasma and serum

**Fig. 3:**
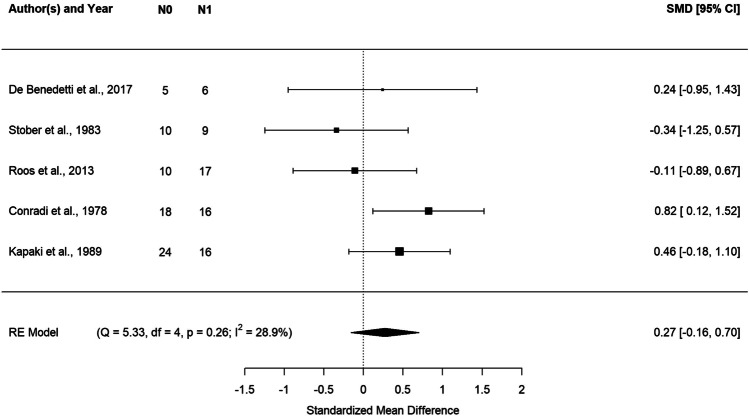
Forest plot: meta-analysis of five studies on plasma/serum samples. Each study is represented with a square centered at the point estimate of the standardized mean difference, with a horizontal line (whiskers) extending on either side of the square, representing the 95% confidence interval of the point estimate. The area of the square is proportional to the corresponding study weight. Combined estimate and 95% confidence interval is represented with a black diamond

Eligible studies on plasma and serum Pb levels were combined for meta-analysis after data standardization to obtain a combined estimate for the two matrices, referred here as plasma/serum*.* The results of the meta-analysis on plasma/serum are described in Fig. 3 via forest plot. We found higher levels of Pb in ALS cases, although the standardized mean difference was not statistically significant. The estimate standardized mean difference was 0.27 with a 95% CI of [− 0.16, 0.70] and *p* value = 0.21. Also, we found some evidence of study heterogeneity looking at the Cochran’s *Q* (*Q* = 5.33, df = 4, *p* = 0.26) and *I*^2^ statistics (*I*^2^ = 28.9%) (Fig. 3).

#### Cerebrospinal fluid Pb in ALS patients and controls

**Fig. 4:**
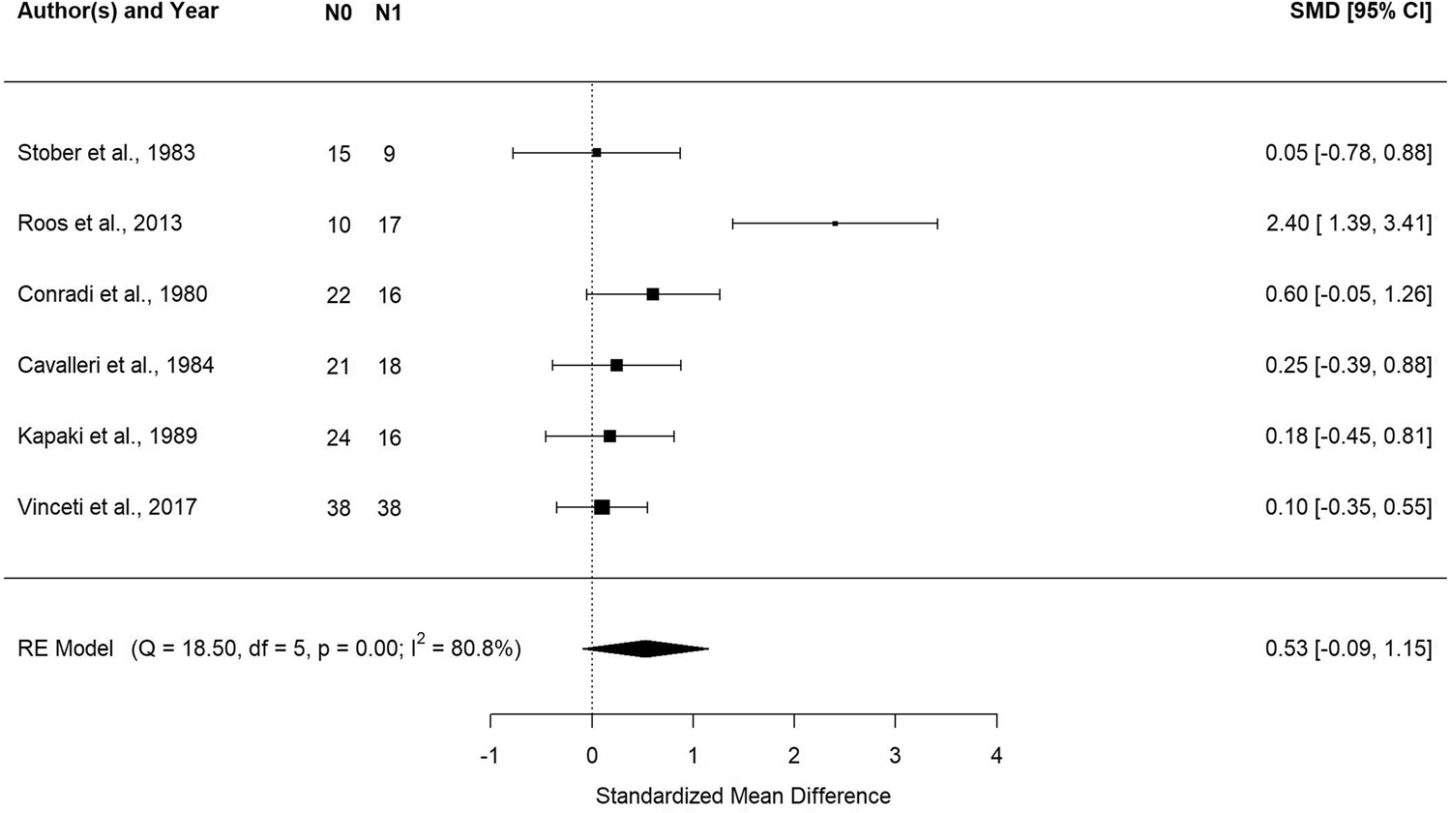
Forest plot: meta-analysis of six studies on CSF samples. Each study is represented with a square centered at the point estimate of the standardized mean difference, with a horizontal line (whiskers) extending on either side of the square, representing the 95% confidence interval of the point estimate. The area of the square is proportional to the corresponding study weight. Combined estimate and 95% confidence interval is represented with a black diamond

The results of the meta-analysis on CSF Pb levels are described in Fig. 4 via forest plot. We found higher values of Pb in ALS cases compared to controls, although the standardized mean difference was borderline statistically significant. The estimate was 0.53 with a 95% CI of [− 0.09, 1.15] and *p* value = 0.09. No evidence of effect heterogeneity was found looking at the Cochran’s *Q* (*Q* = 18.5, df = 5, *p* < 0.0001) and the *I*^2^ statistics (*I*^2^ = 80.8%) (Fig. 4).

### Publication bias and sensitivity analyses

**Fig. 5:**
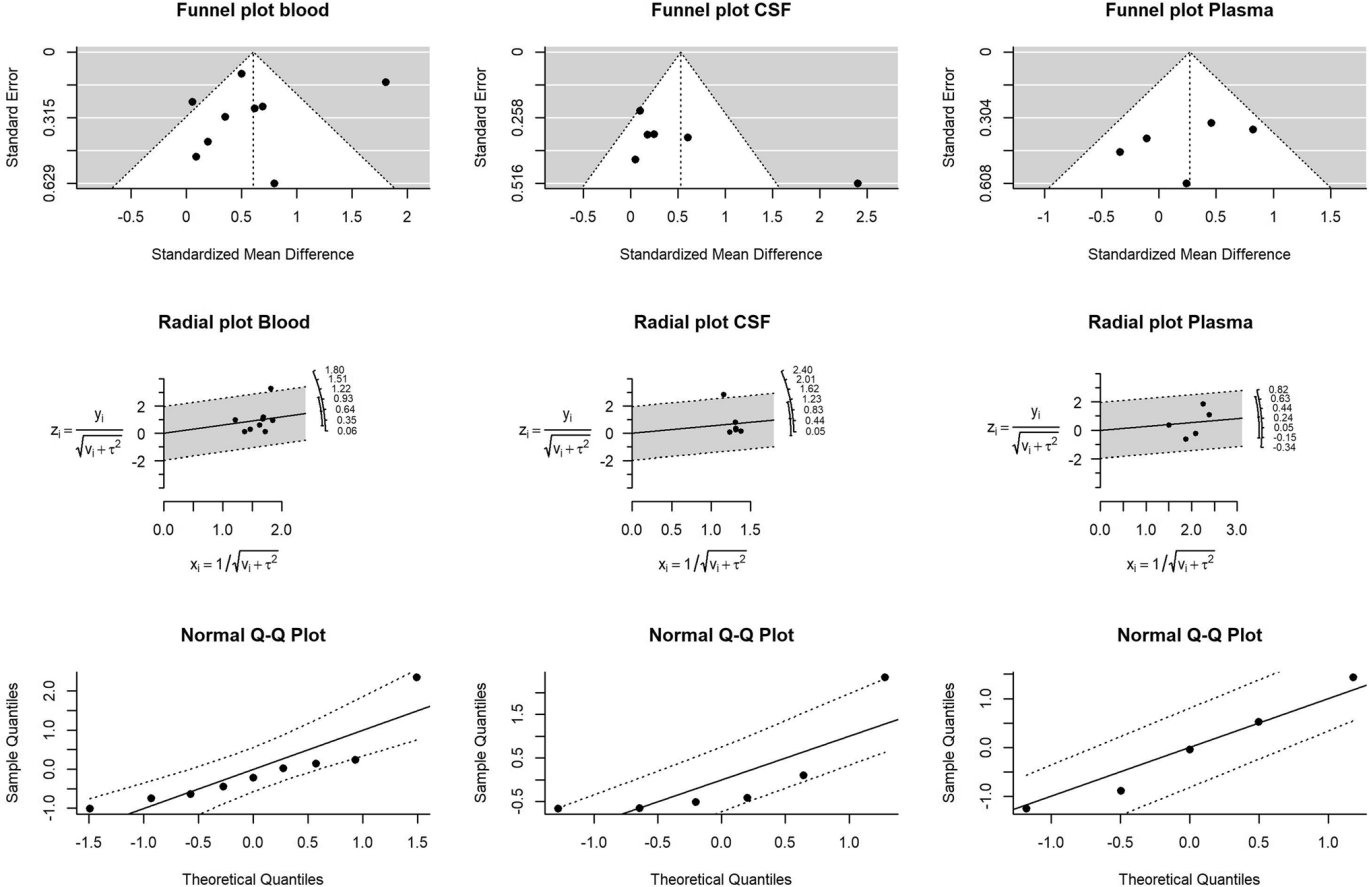
Funnel plot, radial plot, and normal Q-Q plot for whole blood, plasma/serum, and CSF

We have investigated possible publication bias by the analysis of the funnel plot, Galbraith radial plot, and normal quantile–quantile (Q-Q) plot (Fig. 5).

The visual inspection of the funnel plots highlights some degree of asymmetric distribution around the central estimates for the analyses in blood and CSF indicating possible source of publication bias. Specifically, one study estimate in blood and one study estimate in CSF fall on the right side of the “funnel triangles” indicating overestimation of the differences between cases and controls. However, no evidence of publication bias can be observed looking at radial and Q-Q plots.

To avoid any doubt about the robustness of our results in blood, we run a leave-one-out sensitivity analysis, confirming the significant association in blood (Table 1).

**Table 1:** Sensitivity leave-one-out analysis in blood. Table reports standardized mean differences estimated excluding one study at a time. All the sub-analyses lead to a significant association as observed in the main analysis. Excluding the study of Eum et al. leads to a reduction of the effect size but to a lower *p* value (higher statistical significance) due to the reduced heterogeneity. Overall these results confirm the robustness of the main analysis in blood sample

Study	SMD (95% CI)	Meta-analysis *p*
*De Benedetti *et al*. *[15]	0.59 (0.16–1.02)	0.0071
*Sienko *et al*. *[17]	0.65 (0.22–1.08)	0.0028
*Stober *et al*. *[18]	0.65 (0.21–1.08)	0.0035
*Vinceti *et al*. *[19]	0.64 (0.19–1.08)	0.0053
*Garzillo *et al*. *[20]	0.60 (0.14–1.06)	0.0098
*Oggiano *et al*. *[21]	0.59 (0.13–1.05)	0.0111
*Qin *et al*. *[22]	0.69 (0.26–1.12)	0.0016
*Eum *et al*. *[23]	0.45 (0.30–0.61)	< 0.0001
*Fang *et al*. *[16]	0.62 (0.16–1.08)	0.0089

## Discussion

Amyotrophic lateral sclerosis is a neurodegenerative disease that affects motor neurons. Although it can be defined a rare disease, it leads to a significantly lower quality of life of patients and their families, causing progressive overtime immobility that requires total assistance, resolving with the death of the patients in a period that goes from about 24 to 50 months from diagnosis [30].

Despite the huge attention of research on this topic, much of its etiology is not yet known, suggesting that both environmental and genetic factors contribute to its onset and severity [3, 4]. Among the environmental factors, heavy metals have attracted the attention of researchers, in particular Pb, because of its demonstrated neurotoxicity [31–34].

Due to the reasons mentioned above, this meta-analysis work aims to clarify the association of Pb levels in human tissues with the onset of ALS, a link still not clarified despite the numerous publications on the subject. We conducted a systematic research of the works published until February 2022. The works that met our research criteria were fourteen case–control studies for concentrations of Pb in whole blood, plasma/serum, and CSF. Of these works, nine took whole blood samples, five plasma/serum, and six CSF (some are considering more than a tissue).

For all tissues analyzed, we observed a positive trend that shows an association between the concentrations of Pb and the presence of the disease. However, the only tissue that gave a statistically significant result was whole blood (SMD = 0.61; 95% CI 0.20, 1.01; *p* = 0.003).

Although the combined standardized difference between cases and controls was positive (higher in ALS cases) for all three tissues, we observed in serum/plasma two publications in which Pb concentration was lower in ALS cases than controls. The two articles [18, 24] reported the value as a median rather than as a mean and this made necessary their transformation into average values, in order to make them homogeneous and comparable with other articles. This conversion may have played a role in the presence of these anomalous data, in fact in the paper of Roos et al. [24] values which were expressed as a median showed higher values in ALS patients than the controls; however, in both values the difference is really low and without statistical significance.

We obtained significant association in whole blood only. This result can be explained by the small number of studies and patients in other matrices, highlighting the importance of further studies in the field. However, it is crucial to consider the distribution of this metal, showing a particular propensity for cellular structures, within which it tends to accumulate. In particular, Pb tends to accumulate inside the erythrocytes rather than into the plasma component [35–37]. One of the most targeted components in the erythrocytes is the d-aminolevulinic acid dehydratase (ALAD), an enzyme fundamental for the synthesis of heme groups [8, 36]. Another target is the pyrimidine 5′-nucleotidase 1, involved in the pyrimidine metabolism [38]. This could explain why the most consistent results were observed in whole blood compared to plasma/serum, indicating the former as a preferable matrix for this type of analysis. The same considerations apply to the CSF. In fact, this is also an acellular matrix, and several works show how the Pb tends to accumulate within the choroid plexus or to be seized by the glia, specifically in astrocytes, explaining why its concentrations in the CSF may be too low to allow discrimination between patients and controls. On the other hand, it must be considered that at the bone level, one of the main tissues of accumulation of Pb [39, 40], the latter tends to accumulate at the level of the extracellular matrix, thanks to its ability to replace the divalent ions, such as the Ca^2+^, important component for the formation of the bone matrix [41]. The bone tissue represents one of the longest-lived reserves of Pb in the human body, capable of mobilizing such reserves, for example, following periods of prolonged immobility. Such consideration is very important and in the interpretation of the results presented here, since, as described in Vinceti et al., the differences between ALS patients and controls could be linked to the long period of immobilization of patients and the consequent bone resorption followed by the mobilization of Pb reserves. This may introduce a bias in the analyses making the causal link between the blood concentrations of this metal and the onset of the disease difficult to interpret [19, 27].

In support of the eligibility of Pb as an etiological factor in ALS is its marked neurotoxic cytotoxicity, also on a charge of the motor neurons, it has been observed that chronic exposure to this metal is accompanied by gradual atrophy of skeletal muscles [24, 33]. Furthermore, it has been observed that at the origin of the neuropathy following the intoxication by Pb, finds its origin at the level of the anterior horn of the spinal cord [24]. Pb is able to overcome the blood–brain barrier, and its accumulation in choroidal plexuses can cause their alteration resulting in damage to these structures and to the brain, with consequent accumulation in the glia and neurons [7–9]. In addition, changes in the blood–brain membrane are detected in almost 50% of ALS patients [42–44]. At the intracellular level, the mechanisms of action of Pb are known, it exerts its cytotoxicity by altering the homeostasis of the essential elements of the organism and mimes divalent ions such as zinc (Zn^2+^) and Ca^2+^, also binds thiolate proteins (-SH), and as previously seen alters the production of heme groups and induces oxidative stress, also altering systems of protection against this phenomenon [45, 46]. From a molecular point of view, a recent study conducted in mice highlights the ability of Pb to induce the formation of aggregates and the inclusion of TAR DNA-binding protein (TDP-43), one of the main phenomena that occur in motor neurons during the ALS [47], data that although preliminary offer a possible scenario on the etiopathogenesis of Pb at the molecular level. In addition, Pb can alter neuronal transmission by acting on NMDR receptors, acetylcholine receptors, and voltage-dependent calcium channels [37].

Although the present work does not allow to assert a certain correlation between Pb levels in the body and ALS, it positively supports this hypothesis, showing a synergistic enhancement of the literature supporting this thesis and highlighting the usability of Pb levels in whole blood as a biomarker, probably in combination with other factors. Although further studies are necessary in order to increase the statistical power and to better clarify the possible cellular mechanisms underlying the possible role of Pb in the occurrence of ALS, this paper provide a further evidence favoring this hypothesis. Furthermore, as previously discussed, it is not clear if the accumulation of Pb in blood and other biological tissues is the cause or the consequence of the disease making it necessary to clarify this point. Additional epidemiological prospective studies are needed to reduce the risk of misinterpretation due to reverse causation. Besides, the recently developed Mendelian randomization (MR) approach can provide further evidence about causality in this association, as it has recently done to clarify the association of ALS with other traits [48]. As recently pointed out in the review by Julian et al., high-quality genome-wide association studies (GWAS) on lead levels and ALS risk are needed to identify robust instrumental variables for this purpose and avoid incorrect result interpretations [48].
